# Tolerating the “doubting Thomas”: how centrality of religious beliefs vs. practices influences prejudice against atheists

**DOI:** 10.3389/fpsyg.2015.01352

**Published:** 2015-09-08

**Authors:** Jeffrey Hughes, Igor Grossmann, Adam B. Cohen

**Affiliations:** ^1^Department of Psychology, University of WaterlooWaterloo, ON, Canada; ^2^Department of Psychology, Arizona State UniversityTempe, AZ, USA

**Keywords:** anti-atheist prejudice, cultural differences, religion, beliefs, practices

## Abstract

Past research has found a robust effect of prejudice against atheists in largely Christian-dominated (belief-oriented) samples. We propose that religious centrality of beliefs vs. practices influences attitudes toward atheists, such that religious groups emphasizing beliefs perceive non-believers more negatively than believers, while groups emphasizing practices perceive non-practicing individuals more negatively than practicing individuals. Studies 1–2, in surveys of 41 countries, found that Muslims and Protestants (belief-oriented) had more negative attitudes toward atheists than did Jews and Hindus (practice-oriented). Study 3 experimentally manipulated a target individual's beliefs and practices. Protestants had more negative attitudes toward a non-believer (vs. a believer), whereas Jews had more negative attitudes toward a non-practicing individual (vs. a practicing individual, particularly when they had a Jewish background). This research has implications for the psychology of religion, anti-atheist prejudice, and cross-cultural attitudes regarding where dissent in beliefs or practices may be tolerated or censured within religious groups.

## Introduction

Numerous studies have demonstrated prevalent negative attitudes toward atheists (Swan and Heesacker, 2012; Gervais, 2013; Cook et al., 2015). For example, Americans rate atheists as the top group that “does not at all agree with my vision of American society” (39.6%), above other groups that are often targets of prejudice, such as homosexuals (22.6%) and Muslims (26.3%; Edgell et al., 2006). Recent work suggests that prejudice—negative attitudes toward a group (Dovidio and Gaertner, 2010)—toward atheists is largely driven by atheists' lack of belief in a watchful, moralizing God (Gervais et al., 2011; Gervais, 2013). However, research on atheist prejudice has almost universally been conducted in the US, a country with a Protestant majority (and three-quarters Christian; Pew Research Center, 2008); thus, while Christians have shown evidence of anti-atheist attitudes (Jackson and Hunsberger, 1999), it is an open question whether the link between religion and prejudice against atheists—and its underlying processes—would translate to non-Christian samples around the world. This is an important question because not all religious groups may share the American, Christian focus on beliefs.

Religion can be viewed as a form of culture (Snibbe and Markus, 2002; Belzen, 2009; Shariff et al., 2010; Atkinson and Whitehouse, 2011), with systematic cultural differences in the extent religions emphasize beliefs or practices. Protestants place a stronger emphasis on beliefs, whereas other religions, like Judaism, are more practice-oriented (Cohen et al., 2003; Cohen and Hill, 2007). It should be noted that this emphasis on beliefs or practices, at the group level, is distinct from the beliefs or practices to which any individual may adhere (Na et al., 2010; Smaldino, 2014). Drawing from social identity theory (Tajfel and Turner, 1979), this group-level emphasis on beliefs or practices should differentially define who is perceived as a member of the ingroup.

If atheists as a group are largely defined by their lack of belief in God (Swan and Heesacker, 2012), then this lack of belief should be more relevant for religions that place greater importance on believing, and they should be more likely to be perceived as outgroup members. In contrast, practice-oriented religions should place less emphasis on lack of belief as a criteria for ingroup membership. Thus, anti-atheist prejudice may be much lower within practice-oriented religions than what the previous research on US samples would suggest. We examine this distinction in Studies 1 and 2. This contrast may also hold for non-believing individuals who are perceived to be within a religious community. Belief-oriented religions may be more tolerant of a member who fails to participate in certain religious practices, while practice-oriented religions may be more likely to tolerate a non-believing “doubting Thomas” within their midst. We explore this further in Study 3.

Much of the behavioral research concerning religious beliefs and practices has focused its attention on Protestants and Jews. For instance, when making judgments about religiosity, Protestants place more emphasis than Jews on religious beliefs (e.g., belief in God, belief in an afterlife), whereas Jews place more emphasis on religious practices (e.g., attending religious services, reading religious texts; Cohen et al., 2003). Moreover, in practice-oriented (vs. belief-oriented) religions, group affiliation and social connection are seen as integral parts of religious identity (Cohen and Hill, 2007). As a result, practice-oriented religions like Judaism are more likely to stress the importance of heredity or an ethnic identity for one's religious identity, and place an emphasis on religious practices and rituals as a means of maintaining group cohesiveness. The implication of this is that the religious culture in which individuals reside influences how they perceive their own religiosity. In a belief-oriented religion, an individual who does not believe in God may perceive herself as not very religious; in contrast, the same individual in a practice-oriented religion may consider herself as very religious, depending on how well she adheres to common religious practices within that culture. In this sense, the group-level emphasis on beliefs or practices is distinct from individual adherence to beliefs and practices (Na et al., 2010; Smaldino, 2014), but still exerts influence on how individuals' religiosity is perceived by themselves and others.

To date, however, there has been little empirical research on what other major religious groups emphasize. However there is at least some evidence that Muslims, similar to Christians (Cohen and Rozin, 2001), perceive the control of thoughts and intentions to be relevant for moral judgments (Inozu et al., 2012). Conversely, Hindus have been shown to judge even unintentional harmful actions more harshly than Protestants, suggesting an emphasis on acts rather than beliefs (Laurin and Plaks, 2014). While the literature here is sparse, this at least suggests that Muslims may be belief-oriented, while Hindus may be practice-oriented. In the present paper, we extend research to these two major religions and examine these assumptions directly in Study 2.

## Present research

Using nationally representative surveys and experiments, three studies examine attitudes of Protestant Christians (Studies 1–3), Muslims (Study 2), Jews (Studies 1–3), and Hindus (Studies 1–2) toward atheists (Studies 1–2) and individuals who vary in their beliefs and practices (Study 3). Given established differences in cross-cultural religious emphases on beliefs and practices, we hypothesized that predominantly belief-oriented religions (Protestants and Muslims) would have more negative attitudes toward atheists and non-believers. Conversely, predominantly practice-oriented religions (Jews and Hindus) would have less negative attitudes toward atheists, but more negative attitudes toward non-practicing individuals—particularly when those individuals are perceived to be hereditary/ethnic members of that religion.

We also predicted that these results should be moderated by self-assessments of one's own religiosity. Previous research in cultural psychology has found that the link between beliefs and religiosity is stronger for Protestants than for Jews (Cohen et al., 2003). In contrast, Jews' religiosity is most strongly linked with their religious practices. Thus, if Protestants consider religiosity more in terms of beliefs as compared to practices, then strongly religious (vs. less religious) Protestants should show more negative attitudes toward atheists (non-believers); in contrast, attitudes of members of predominantly practice-oriented religions like Judaism and Hinduism toward atheists should not be as strongly influenced by religiosity. Instead, religiosity should moderate the attitudes of members of practice-oriented religions toward non-practicing individuals.

## Study 1

Study 1 provided an initial test of our hypothesis that attitudes toward atheists would differ by religious group, using feeling thermometer measures common in the prejudice literature (e.g., Dasgupta and Greenwald, 2001; Gervais et al., 2011). We also examined political conservatism as a potential confound, and assessed the moderating role of religiosity for these religious differences.

### Method

#### Sample

We recruited 100 American Protestants, 56 American Jews, and 150 Indian Hindus from Amazon Mechanical Turk for a study on “attitudes toward social groups” (see Table 1 for demographics). Sample size was determined in advance, and we aimed for 100–150 participants for each religious group. However, before examining the results, we ended collection from Jewish participants early due to slow participation rates. Participants were screened for their religion at the beginning of the study. This study was carried out in accordance with the recommendations of the Office of Research Ethics at the University of Waterloo, with informed consent from all participants in accordance with the Declaration of Helsinki.

**Table 1:** **Demographics for studies 1–3**.

	***N***	**% Women**	**Age**	**% Completed college**	**Religiosity**	**Political conservatism**	**Fundamentalism**
**STUDY 1**							
Protestants	100	50.0%	38.2 (14.8)	58.0%	4.84 (1.71)	4.4 (1.91)	–
Jews	56	41.1%	31.8 (12.2)	63.6%	4.37 (1.79)	3.75 (1.84)	–
Hindus	150	37.3%	30.7 (9.6)	89.3%	5.23 (1.46)	4.21 (1.61)	–
**STUDY 2**							
Protestants	12,188	57.7%	48.7 (17.4)	34.8%	4.43 (1.35)	–	0.16 (0.36)
Muslims	2167	50.6%	39.7 (14.7)	16.4%	5.37 (1.22)	–	0.50 (0.50)
Jews	1104	56.5%	45.2 (17.9)	40.0%	4.02 (1.61)	–	0.46 (0.50)
Hindus	203	51.7%	43.0 (16.5)	26.6%	5.26 (1.11)	–	0.11 (0.31)
**STUDY 3**							
Protestants	311	42.8%	–	–	–	–	–
Jews	271	45.8%	–	–	–	–	–

*Due to a technical error, age, and education were not collected for Study 3. Means and standard deviations (in parentheses) are displayed for age, religiosity, political conservatism, and fundamentalism*.

#### Procedure

To provide a general measure of prejudice, participants first rated atheists on a “feeling thermometer” from 0 to 100. This measure was mixed in with feeling thermometers for several other distractor groups, to make the critical measure less overt, as well as one measuring their feelings toward “people in general.” Next, participants rated the same groups on measures of distrust and disgust, on scales from 0 (very untrustworthy or very disgusting) to 100 (very trustworthy or very pleasant). All items were then reverse-coded so high scores meant more negative feelings, more distrust, and more disgust. A principal component analysis supported a one-factor solution (67.51% variance explained; all component loadings above 0.69); thus, all three items were combined into a composite score of overall negative attitudes (Cronbach's α = 0.76). Finally, participants completed demographics, including a measure of political beliefs (from 1 = “liberal” to 7 = “conservative”) and a measure of religiosity (“How religious and/or spiritual are you?” from 1 = “not at all” to 7 = “very”; see Table 2 for correlations between variables for all studies)^1^.

**Table 2:** **Correlation tables for studies 1–3**.

	**Mean**	**SD**	**1**	**2**	**3**	**4**	**5**	**6**	**7**	**8**	**9**	**10**	**11**
**STUDY 1**													
1. Jewish (d)	0.18	0.39	–										
2. Hindu (d)	0.49	0.50	**−0.46**	–									
3. Atheist neg. feelings	46.82	20.76	−0.05	**−0.14**	–								
4. General neg. feelings	34.45	16.86	0.10	**−0.17**	**0.30**	–							
5. Male (d)	0.57	0.50	−0.02	0.10	0.01	**0.14**	–						
6. Age	33.35	12.41	−0.06	**−0.21**	0.10	**−0.15**	−0.10	–					
7. Religiosity	4.95	1.63	**−0.16**	**0.17**	0.06	**−0.26**	0.00	**0.14**	–				
8. Political conservatism	4.19	1.76	**−0.12**	0.01	0.09	−0.06	0.08	**0.12**	**0.36**	–			
STUDY 2^*^													
1. Jewish (d)	0.07	0.26	–										
2. Hindu (d)	0.01	0.11	**−0.03**	–									
3. Muslim (d)	0.14	0.35	**−0.11**	**−0.05**	–								
4. Believes in God (d)	0.82	0.38	**0.02**	**0.04**	**0.17**	–							
5. Religious attendance	4.21	2.74	**−0.05**	0.01	**0.24**	**0.39**	–						
6. Atheist neg. feelings	3.07	1.22	**−0.05**	**−0.07**	**0.23**	**0.22**	**0.27**	–					
7. Male (d)	0.43	0.50	0.00	0.01	**0.05**	**−0.11**	**−0.04**	0.00	–				
8. Age	47.11	17.39	**−0.03**	**−0.03**	**−0.17**	0.01	**−0.03**	−0.01	0.01	–			
9. Education	2.66	1.58	**0.09**	0.00	**−0.15**	**−0.13**	**−0.11**	**−0.17**	**0.05**	**−0.17**	–		
10. Religiosity	4.55	1.40	**−0.10**	**0.06**	**0.24**	**0.59**	**0.54**	**0.26**	**−0.09**	**0.03**	**−0.15**	–	
11. Fundamentalist (d)	0.23	0.42	**0.15**	**−0.03**	**0.27**	**0.22**	**0.35**	**0.26**	−0.01	**−0.08**	**−0.16**	**0.31**	–
**STUDY 3**													
1. Participant Jewish (d)	0.47	0.50	–										
2. Target Jewish (d)	0.51	0.50	−0.01	–									
3. Target believes (d)	0.49	0.50	0.00	−0.06	–								
4. Target practices (d)	0.48	0.50	0.01	−0.01	−0.02	–							
5. Neg. feelings	36.98	22.01	0.02	−0.08	**−0.31**	**−0.09**	–						
6. Male (d)	0.56	0.50	−0.03	−0.01	0.02	−0.02	0.04	–					

*Bolded values are significant at the p < 0.05 level. Variables followed by “(d)” are dummy-coded (i.e., 0 or 1)*.

* *Correlations in Study 2 shown here do not take into account country-level variation and thus should be interpreted with caution*.

#### Results

**Figure 1:**
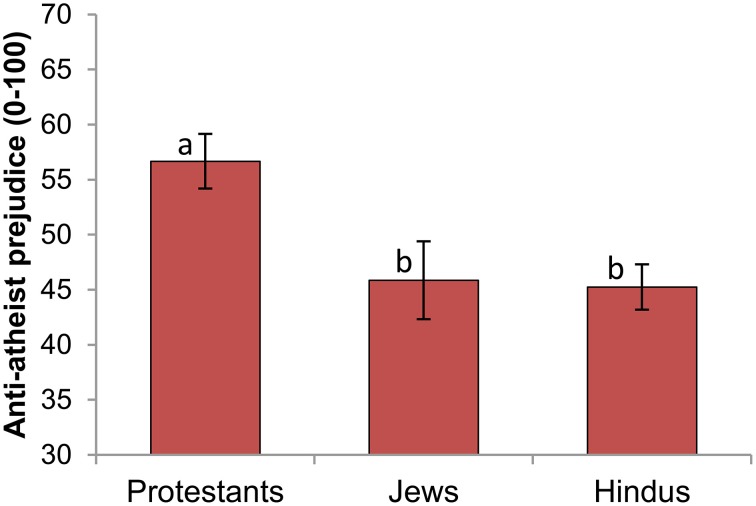
**Negative feelings toward atheists, by participants' religious group membership**. Error bars indicate ±1 SE. Different subscripts indicate that these bars differ from each other at the *p* < 0.05 level.

Our primary prediction was that Protestants would report higher levels of prejudice toward atheists than would Jews or Hindus. To control for individual or group differences in general positivity/negativity toward others, we included participants' feeling thermometer score for “people in general” as a covariate. An ANCOVA revealed a significant effect of religious background, *F*_(2, 292)_ = 4.00, *p* = 0.02, η^2^ = 0.027. These results were significant and in the same direction without including the covariate, *F*_(2, 293)_ = 5.26, *p* = 0.006, η^2^ = 0.036. As Figure 1 indicates, Protestants had more negative feelings toward atheists (*M* = 52.21, *SE* = 2.29) than did Jews (*M* = 44.41, *SE* = 2.92), *t*_(148)_ = −2.00, *p* = 0.048; and Hindus (*M* = 43.95, *SE* = 1.47), *t*_(244)_ = −3.16, *p* = 0.002; while Jews and Hindus did not differ from each other, *t*_(194)_ = −0.15, *p* = 0.88. These results also held when controlling for participants' political conservatism, *F*_(2, 287)_ = 3.15, *p* = 0.04, η^2^ = 0.022.

Religiosity significantly moderated the effect of religious group, group × religiosity interaction: β = 0.33, *t*_(286)_ = 2.85, *p* = 0.005. A weighted contrast comparing belief-oriented religions (Protestants) to practice-oriented religions (Jews and Hindus) showed that more religious Protestants showed more prejudice toward atheists than did less religious Protestants, β = 0.34, *t*_(286)_ = 3.68, *p* < 0.001. However, religiosity did not predict prejudice among members of practice-oriented religions, β = 0.01, *t*_(286)_ = 0.13, *p* = 0.90. At high levels of religiosity (+1 SD), members of belief-oriented religions displayed significantly more prejudice toward atheists than members of practice-oriented religions, β = 0.59, *t*_(286)_ = 3.67, *p* < 0.001, but not at low levels of religiosity (−1 SD), β = −0.06, *t*_(286)_ = −0.38, *p* = 0.71 (see Supplementary Material for more detailed analyses).

### Discussion

The results of Study 1 show that Protestants, who place greater emphasis on beliefs relative to Jews and Hindus according to previous research, had more negative attitudes toward atheists. This is initial evidence that the emphasis a religious group places on beliefs or practices may influence prejudice toward non-believers. These results held even after controlling for baseline differences in liking for people in general. The differences in attitudes were also not due to differences in political conservatism, which has been previously linked with anti-atheist prejudice (Edgell et al., 2006) and prejudice more broadly (Terrizzi et al., 2010). In addition, the results of Study 1 provide preliminary evidence that religiosity may moderate these results: for belief-oriented religions, religiosity predicted more negative attitudes, whereas for practice-oriented religions, religiosity had no effect. We test this interaction further in Study 2.

## Study 2

In Study 2, we aimed to replicate and extend Study 1 results using the 2008 International Social Survey Programme: Religion III (ISSP; ISSP Research Group, 2012), an international dataset with nationally representative samples across 41 countries (see Table S1). This allows us not only to extend our findings beyond the US and India, but also to include a large sample of Muslims, as a second belief-oriented religion along with Protestants.

We also attempted to offer more direct empirical evidence that the religious groups under study differ in their emphasis on beliefs and practices. Past research on cross-religious differences has shown that such group-level differences are revealed in the extent to which beliefs and practices predict self-reported religiosity (Cohen et al., 2003; Cohen and Hill, 2007). In essence, religious group members assess their own religiosity by evaluating how much their beliefs and practices accord with the beliefs and practices of their religious group. The aggregation of these assessments offers an indication of the group-level emphasis of beliefs and practices. For belief-oriented religions, their beliefs–religiosity association should be higher than their practices–religiosity association; for practice-oriented religions, their practices–religiosity association should be higher than their beliefs–religiosity association. Thus, to directly test the emphases of these religious groups, we examined how beliefs and practices predicted self-reported religiosity, and whether these relationships were moderated by religious group.

Finally, we attempted to rule out a potential alternative explanation concerning differences in religious fundamentalism between religious groups, which has been previously linked with prejudice toward a variety of stigmatized groups (Altemeyer and Hunsberger, 1992; Hunsberger et al., 1999; Laythe et al., 2001, 2002).

### Method

We screened for participants who identified as being Protestant (*n* = 12,188), Muslim (*n* = 2167), Jewish (*n* = 1104), or Hindu (*n* = 203; see Table 1 for demographics).

As a measure of attitudes toward atheists, we used one item, “What is your personal attitude toward members of the following religious groups? Atheists or non-believers” (1 = “very positive” to 5 = “very negative”).

To measure belief in God, one item was used, “What best describes your beliefs about God?” with four response options: “I don't believe in God and I never have,” “I don't believe in God now, but I used to,” “I believe in God now, but I didn't used to,” and “I believe in God now and I always have.” These were combined into two options indicating participants' present beliefs (0 = “currently does not believe” and 1 = “currently believes”). For religious attendance, one item was used, “How often do you visit a holy place for religious reasons such as going to [shrine/temple/church/mosque]?” (1 = “Never” to 9 = “Several times a week”). These variables were used to predict self-reported religiosity, measured with one item, “Would you describe yourself as…1 = extremely non-religious to 7 = extremely religious.”

To address the possibility that the association between religious group and atheist attitudes was a result of differences in religious fundamentalism, we examined responses to one item asking participants whether truth is found in only one religion (1) or in many religions (0), one important aspect of fundamentalism (Altemeyer and Hunsberger, 1992).

In the analyses for Study 2, we used a multilevel random intercepts model with participants (Level 1) nested in countries (Level 2) to control for between-country effects not associated with religious group. Religious group was then dummy-coded, with Muslims as the comparison group. Because of differing sample sizes between groups leading to different within-group variances, we used a diagonal covariance matrix to model heterogeneous variance (Pinheiro and Bates, 2000). For assessing how beliefs and practices predicted religiosity, we standardized the measures of beliefs and practices, and modeled them with a mixed effects analysis, so we could directly compare the slopes of beliefs and practices in predicting religiosity, and whether religious group moderated these comparisons.

### Results

#### Beliefs, practices, and religiosity

As results in Table 3 show, Muslims and Hindus were most likely to report belief in God, while Protestants were least likely. Muslims had the highest level of religious attendance, while Jews had the lowest.

**Table 3:** **Belief in/importance of God and attendance of religious services, by religious group (Study 2)**.

	**Belief in God (0–1)**	**Religious attendance (1–9)**
Protestants	0.78_a_ (0.41)	3.97_a_ (2.57)
Muslims	0.97_b_ (0.17)	5.87_b_ (3.15)
Jews	0.85_c_ (0.36)	3.74_c_ (2.66)
Hindus	0.95_b_ (0.22)	4.40_a_ (2.48)

*Values indicate group means, with standard deviation in parentheses. Subscripts for each column indicate that these groups differed from each other, as indicated by a Dennett's modified Tukey-Kramer (DTK) multiple comparisons test, which accounts for uneven sample sizes and variances*.

**Figure 2:**
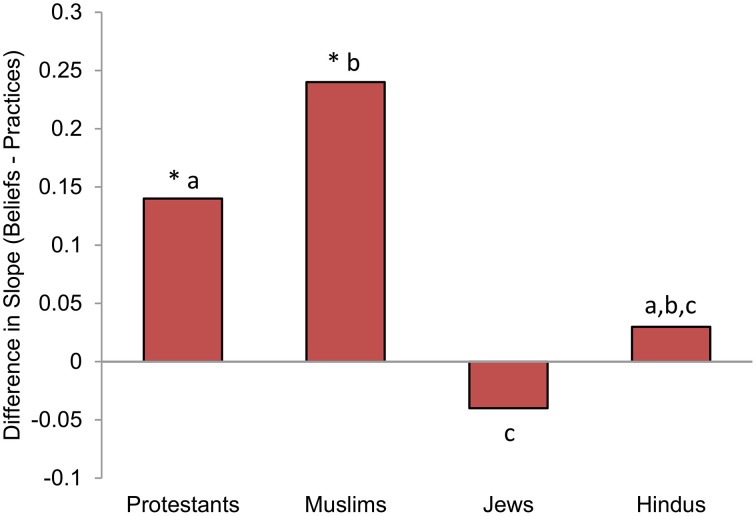
**Differences in slopes for beliefs predicting religiosity and practices predicting religiosity, by religious group**. Asterisks indicate that the difference in slopes is greater than zero. Different subscripts indicate that these bars differ from each other at the *p* < 0.05 level.

We found a significant three-way interaction between religion, item (dummy code indicating beliefs or practices), and value (within-person, continuous measure of beliefs/practices), *F*_(3, 28128)_ = 6.20, *p* < 0.001, *R*^2^ = 0.283 (Lefcheck, 2013; Nakagawa and Schielzeth, 2013). Within each religion, the two-way interactions showed that Protestants placed more emphasis on beliefs than on practices, β = 0.14, *t*_(28128)_ = 12.68, *p* < 0.001; as did Muslims, β = 0.24, *t*_(28128)_ = 5.01, *p* < 0.001. In contrast, Jews placed equal emphasis on beliefs and practices, β = −0.04, *t*_(28128)_ = −0.78, *p* = 0.43; as did Hindus, β = 0.04, *t*_(28128)_ = 0.34, *p* = 0.73 (see Figure 2 and Table 4).

**Table 4:** **Beliefs and practices predicting religiosity, and negative attitudes toward atheists (Study 2)**.

	**Belief in God (slope predicting religiosity)**	**Religious attendance (slope predicting religiosity)**	**Religiosity**	**Negative attitudes toward atheists**
Protestants	0.57	0.43	4.43 (1.35)	2.96 (1.13)
Muslims	0.50	0.26	5.27 (1.22)	3.68 (1.40)
Jews	0.65	0.69	4.02 (1.61)	2.34 (0.92)
Hindus	0.36	0.33	5.26 (1.11)	2.46 (1.36)

*Slopes for belief in God and religious attendance are standardized coefficients of each variable predicting religiosity (1–7 scale). Values for religiosity and negative attitudes toward atheists are group means, with standard deviation in parentheses*.

In addition to this analysis, we used a weighted contrast to further account for differing sample sizes. This contrast compared Muslims and Protestants as a group to Jews and Hindus as a group. When taken together, belief-oriented religions placed greater emphasis on beliefs over practices, while practice-oriented religions did not; interaction: β = 0.18, *t*_(28128)_ = 3.90, *p* < 0.001.

#### Attitudes toward atheists

Replicating Study 1, we found a significant overall effect of religious group on attitudes toward atheists, *F*_(3, 8236)_ = 17.26, *p* < 0.001, *R*^2^ = 0.017. As shown in Table 4, Muslims had more negative attitudes toward atheists compared to Jews, β = −0.50, *t*_(8236)_ = −3.67, *p* < 0.001, and Hindus, β = −0.64, *t*_(8236)_ = −5.76, *p* < 0.001, but did not differ from Protestants, β = −0.07, *t*_(8236)_ = −1.13, *p* = 0.26. Jews had less negative attitudes than Protestants, β = 0.42, *t*_(8236)_ = 3.48, *p* < 0.001, as did Hindus, β = 0.57, *t*_(8236)_ = 6.13, *p* < 0.001, but Jews and Hindus did not differ from each other, β = −0.14, *t*_(8236)_ = −0.94, *p* = 0.35. The same pattern of results held when simultaneously controlling for age, gender, and education, *F*_(3, 8158)_ = 16.88, *p* < 0.001, *R*^2^ = 0.029. The pattern and significance of results also held when excluding participants who indicated they did not believe in God, *F*_(3, 5917)_ = 15.73, *p* < 0.001, *R*^2^ = 0.020. See also the Supplementary Material for additional analyses regarding Catholics.

#### Effect of fundamentalism

Although fundamentalism itself predicted more negative attitudes toward atheists, *F*_(1, 7561)_ = 128.43, *p* < 0.001, adding this variable as a fixed effect did not change the pattern of results for religious group, *F*_(3, 7561)_ = 17.01, *p* < 0.001, *R*^2^ = 0.030.

#### Moderation of religiosity

Finally, we assessed whether religiosity moderated the effects of religious group on attitudes toward atheists. The overall interaction was not significant, *F*_(3, 8054)_ = 1.43, *p* = 0.23, *R*^2^ = 0.042. However, the weighted contrast comparing Muslims and Protestants together to Jews and Hindus together was marginally significant, β = 0.16, *t*_(8054)_ = 1.74, *p* = 0.08, though the effect size was small. Religiosity predicted more negative attitudes for Muslims and Protestants, β = 0.16, *t*_(8054)_ = 13.43, *p* < 0.001; however, it was not predictive of attitudes for Jews and Hindus, β < 0.001, *t*_(8054)_ = 0.01, *p* = 0.996.

### Discussion

The results from Study 2 replicate those of Study 1 on a large-scale, representative sample from a wide range of countries. This indicates the robustness of the effect, wherein Muslims and Protestants, who place greater emphasis on beliefs relative to Jews and Hindus, report more negative attitudes toward atheists.

This study also rules out a potential alternative explanation for the results. We found that the pattern of results held when controlling for the belief that only one religion holds truth, suggesting that the results are not simply due to differing levels of religious fundamentalism between religious groups.

Finally, Study 2 also provides direct empirical support for the notion that religious groups differ in their emphasis on beliefs and practices. Although such differences have been shown between Protestants and Jews (Cohen et al., 2003; Cohen and Hill, 2007), this study is the first to demonstrate comparable differences for Muslims and Hindus as well. Although we cannot get a direct measure of group-level cultural differences in the centrality of beliefs and practices, we used the association between beliefs, practices, and religiosity to examine how group members use beliefs and practices to assess their own religiosity, i.e., to assess their cultural “fit.” By aggregating these assessments via regression, we capture the group-level emphasis of beliefs and practices.

In addition, religiosity moderated the effects of religious group on anti-atheist prejudice, leading to more negative attitudes in belief-oriented religions but not influencing attitudes in practice-oriented religions. Although, the effect size was small, the fact that religiosity had no impact on attitudes for Jews and Hindus suggests that greater religiosity in a practice-oriented tradition does not result in more negative attitudes toward atheists.

Taken together, the analyses on religiosity provide further support to the idea that that it is the *culture* of these religious groups and their differing emphases on beliefs and practices that are responsible for the differences in anti-atheist prejudice.

## Study 3

Studies 1–2 cumulatively suggest that Protestants and Muslims, who place greater emphasis on beliefs relative to Jews and Hindus, have more negative attitudes toward atheists. In Study 3, we wanted to directly examine the information that members of religious groups use to evaluate a target individual, to further strengthen our argument that beliefs and practices are the critical ingredients. Thus, we experimentally manipulated the beliefs and practices of a target individual to assess their influence on the attitudes of religious individuals toward that target. Study 3 also avoided the use of the term “atheist,” to avoid stereotypic reactions to the label itself (Swan and Heesacker, 2012).

We predicted that Protestants would have less negative attitudes toward a Christian believer (vs. non-believer). In contrast, Jews would have less negative attitudes toward a practicing (vs. non-practicing) Jew.

### Method

#### Sample

We recruited 311 American Protestants and 271 American Jews from Mechanical Turk for a study about “first impressions of individuals” (see Table 1 for demographics). Sample size was determined in advance, and we aimed for 300 participants for each religious group. As in Study 1, participants were screened for their religion at the beginning of the study. This study was carried out in accordance with the recommendations of the Office of Research Ethics at the University of Waterloo, with informed consent from all participants in accordance with the Declaration of Helsinki.

#### Procedure

Participants first read a short description about a target individual who varied between-subjects on three dimensions: religious upbringing (Christian vs. Jewish), beliefs (does vs. does not believe), and practices (does vs. does not practice). Care was taken to select beliefs and practices which were appropriate and relevant for each religion, based upon pilot testing. The believing and practicing target [Christian/Jewish] was described as follows:

Ruth grew up in a [Christian/Jewish] home. She is a teacher at an elementary school in a large American city. She enjoys the process of developing teaching plans, but also appreciates the chance to help students learn and develop. When asked about her religious beliefs, Ruth says that she believes in God, in an afterlife, and believes that [Jesus died and rose again / the Torah came from God]. She attends her local [church/synagogue] regularly, and participates in all major [Christian/Jewish] holidays.

The descriptions for the non-believing targets listed the same beliefs, but indicated instead that the target did not believe them. Similarly, the descriptions for the non-practicing targets listed the same practices, but indicated that the target did not practice them. Subsequently, participants were asked five questions on a scale from 0 to 100: how they felt toward Ruth in general, how much they liked Ruth, how similar they and Ruth were, how trustworthy Ruth was, and how disgusting/pleasant Ruth was. These measures were reverse-coded so that higher scores indicated more negative attitudes.

In contrast to Study 1 results, a principal components analysis of the five attitude items revealed that the measure of disgust very clearly loaded on a separate factor from the remaining four items (first factor eigenvalue = 3.23, 64.55% variance explained; second factor eigenvalue = 1.00, 19.99% variance explained; all items had loadings above 0.81 on their respective components). One notable difference between Study 1 and Study 3 was the change in target from a group to an individual. Participants may have found it less intuitive to evaluate “how disgusting is Ruth” compared to “how disgusting are atheists” as a group. However, the first four attitude items (feelings, liking, similarity, and trust) showed high reliability (α = 0.91), and hence were combined into a score of general attitudes. We examined the measure of disgust separately (see results in Supplementary Material).

### Results

We predicted two Three-Way interactions, and two factorial ANOVAs revealed the predicted interactions: participant religion × target religion × target beliefs, *F*_(1, 572)_ = 5.87, *p* = 0.02, η^2^ = 0.009; and participant religion × target religion × target practices, *F*_(1, 572)_ = 4.13, *p* = 0.04, η^2^ = 0.007. Given our primary interest in participant group differences, we then analyzed the corresponding two-way interactions separately for Protestants and Jews.

**Figure 3:**
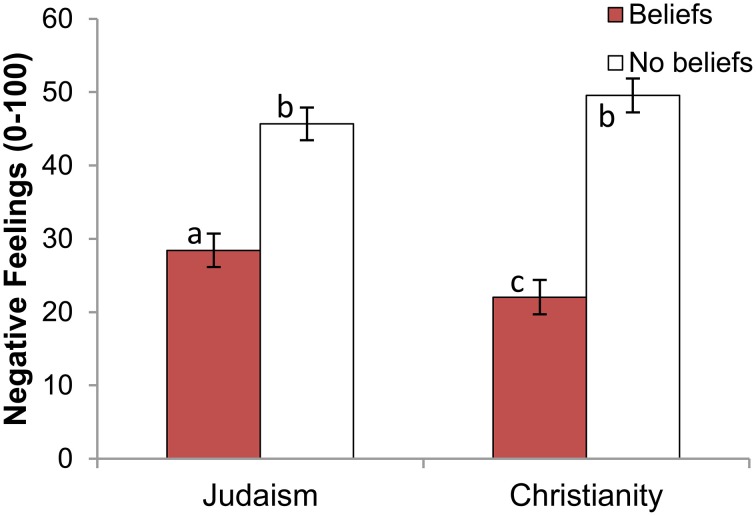
**Negative feelings toward target, by target religion and target level of beliefs (Protestant participants only)**. Error bars indicate ±1 SE. Different subscripts indicate that these bars differ from each other at the *p* < 0.05 level.

For Protestants, we found a two-way target religion × target *beliefs* interaction, *F*_(1, 306)_ = 5.01, *p* = 0.03, η^2^ = 0.012; however, the two-way target religion × target *practices* interaction was not significant for Protestants, *F*_(1, 306)_ = 0.41, *p* = 0.52, η^2^ = 0.001. As Figure 3 indicates, Protestants felt less negatively toward a Christian who believed than one who did not believe, *F*_(1, 148)_ = 63.51, *p* < 0.001, η^2^ = 0.300 (see Table 5). They similarly rated a believing Jew less negatively than a non-believing Jew, *F*_(1, 158)_ = 32.17, *p* < 0.001, η^2^ = 0.169, though this effect was about half the magnitude.

**Table 5:** **Negative attitudes and disgust toward target individual (Study 3)**.

	**Christian target**		**Jewish target**	
	**Beliefs**	**No beliefs**	**Beliefs**	**No beliefs**
**NEGATIVE ATTITUDES**				
**Protestant participants**				
Practices	21.17 (3.17)	43.96 (3.17)	27.96 (3.54)	41.24 (3.34)
No practices	23.07 (3.44)	55.79 (3.34)	28.76 (2.96)	49.15 (2.96)
**Jewish participants**				
Practices	42.20 (3.93)	45.43 (3.49)	26.21 (3.39)	32.80 (3.30)
No practices	40.09 (2.92)	39.67 (3.79)	31.87 (4.18)	39.78 (3.13)
**DISGUST**				
**Protestant participants**				
Practices	34.15 (5.83)	45.00 (5.83)	38.88 (6.52)	52.22 (6.15)
No practices	55.79 (6.33)	47.72 (6.15)	52.04 (5.44)	50.67 (5.44)
**Jewish participants**				
Practices	53.73 (7.24)	49.94 (6.42)	48.06 (6.24)	46.35 (6.07)
No practices	57.32 (5.38)	47.86 (6.97)	35.96 (7.69)	57.02 (5.76)

*Values indicate group means, with standard deviation in parentheses*.

**Figure 4:**
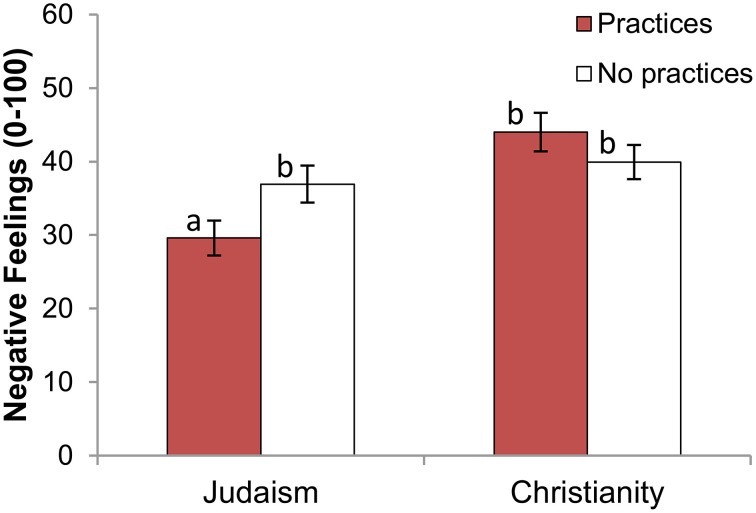
**Negative feelings toward target, by target religion and target level of practices (Jewish participants only)**. Error bars indicate ±1 SE. Different subscripts indicate that these bars differ from each other at the *p* < 0.05 level.

For Jews, the opposite pattern occurred. The two-way target religion × target *beliefs* interaction was not significant, *F*_(1, 266)_ = 1.51, *p* = 0.22, η^2^ = 0.005; but the two-way target religion × target *practices* interaction was significant, *F*_(1, 266)_ = 5.37, *p* = 0.02, η^2^ = 0.019. As Figure 4 indicates, Jews felt less negatively toward a Jew who practiced than one who did not, *F*_(1, 134)_ = 4.41, *p* = 0.04, η^2^ = 0.032 (see Table 5); however, they did not rate practicing and non-practicing Christians any differently, *F*_(1, 132)_ = 1.38, *p* = 0.24, η^2^ = 0.010.

### Discussion

In Study 3, we found evidence that both participants' and targets' levels of beliefs and practices influence attitudes toward those targets. Protestants had less negative attitudes toward people who believed (especially if they were Christian) than those who did not, whereas Jews had less negative attitudes toward Jews who practiced than those who did not.

These patterns reveal two key points. First, there is evidence that Protestants used information about target beliefs in order to rate attitudes toward her, whereas Jews instead used information about target practices. Second, while Protestants' attitudes were more influenced by target beliefs when she was Christian (vs. Jewish), in general Protestants used target beliefs to evaluate her regardless of group membership. In contrast, Jews only evaluated the target on the basis of practices if she was also Jewish, suggesting a stronger role of heredity or ethnic identity for Jews.

## General discussion

Previous research has shown clear evidence of prejudice against atheists in US samples (Edgell et al., 2006; Swan and Heesacker, 2012; Gervais, 2013). Given the social importance of these effects, assessing how religiosity (including both beliefs and practices) influences these evaluations is vital. Across three studies, we showed how religious groups' emphasis on beliefs vs. practices influences attitudes toward atheists (Studies 1–2), as well as non-believing and non-practicing individuals more generally (Study 3). Muslims and Protestants had more negative attitudes toward atheists than did Jews or Hindus. Protestants also had more positive attitudes toward believing vs. non-believing Christians, whereas Jews had more positive attitudes toward practicing vs. non-practicing Jews. Study 2 demonstrated that these religions differ in the extent to which beliefs or practices are emphasized, and thus participants' own religiosity moderated their attitudes toward atheists. This suggests that these varying attitudes are a result of the match between a target's beliefs and practices and the cultural emphasis of a participant's own religious tradition.

Currently, the preferred explanation for anti-atheist prejudice relies on atheists being inherently untrustworthy because they do not fear divine retribution (Gervais, 2013). In contrast, the present work builds on social identity theory (Tajfel and Turner, 1979), which would suggest that the emphasis on beliefs vs. practices in a religious group differentially defines ingroup members. If beliefs are used in belief-oriented religions to determine who belongs in the ingroup, then atheists may not only be perceived as part of the outgroup, but may also be particularly threatening to the extent that they challenge the shared values and beliefs that define the ingroup itself (Ritter and Preston, 2011; Cook et al., 2015). Thus, one area for future research could examine how identification with the ingroup moderates the link between belief-oriented religions and anti-atheist prejudice.

One interesting note about Study 3 is that Protestants evaluated both Christians and Jews on the basis of their beliefs. In contrast, Jews only used practices to evaluate the person when she was Jewish. One possibility is that for Christians, endorsing the correct set of core beliefs overrides religious background. In contrast, Jews may be more likely to perceive engaging in the right practices as a marker for their cultural identity, applying such a standard only to those with a Jewish background. This is consistent with previous research that shows that Jews are more likely to emphasize heredity or an ethnic identity, marked by particular rituals (Cohen and Hill, 2007). It would also be consistent with research on ideology- vs. heritage-based identity, which distinguishes between identity based on transcendent and abstract values vs. traits and cultural traditions (Ditlmann et al., 2011). Another important extension of this research, then, would be examining how belief- and practice-oriented religions differ in their evaluation of other religious groups, and to what extent they perceive them as part of the ingroup.

It is also interesting to note that despite the group-level emphases by Protestants and Muslims on beliefs, and by Jews and Hindus on practices, these do not necessarily result in reports of greater belief in God by individual Protestants and Muslims, nor do they necessarily result in more religious attendance by individual Jews and Hindus. As previous work has shown, group- or cultural-level processes do not always align with processes at the individual level (Na et al., 2010; Smaldino, 2014). Future, research should examine when and how the culture of a religious group exerts influence on an individual's attitudes and behaviors. This process could be moderated by numerous factors, including the aforementioned identification with the ingroup. It could also be influenced by other prevailing cultural identities (e.g., racial or ethnic identity, political identification) or more local norms (e.g., the beliefs of other members at one's church or mosque).

### Limitations

Although this research provides nuance to the previous research showing prevalent negative attitudes toward atheists, it is not without its limitations. First, in several cases the studies used single-item measures (e.g., religiosity, fundamentalism) that preclude the calculation of reliability. This limitation is at least somewhat mitigated by the items' face validity. Also of note, our primary measures of attitudes toward atheists in Studies 1 and 3 were composite measures of multiple items. However, future studies should better assess the moderating relationship of religiosity using a more comprehensive scale to provide further evidence that religiosity moderates the association between religious group and attitudes toward atheists.

Second, our evidence for differing emphases on beliefs and practices in Study 2 was limited by the use of a dichotomous measure of beliefs. This methodological limitation is a result of using secondary data analysis for which we had no control over the item wording. The use of this dichotomous measure serves to reduce the variance and, thus, the power. The fact that we still found our predicted results despite this methodological limitation is encouraging. However, future studies should examine the relationships between beliefs, practices, and religiosity using continuous measures and, ideally, multiple-item composite measures.

## Conclusion

Our research underscores the importance of accounting for the culture-specific meaning of religiosity when examining religion-related outcomes. This insight has numerous implications. First, the criteria religious groups use to define membership may influence how accepting they are toward outsiders: how such lines are drawn may have ramifications for religious intergroup hostility, of which negative attitudes are the first step (Allport, 1954; Duckitt, 2003). Second, criteria for membership may influence what behaviors are acceptable or unacceptable within the religious community itself. A religious group emphasizing beliefs may be more tolerant toward deviance in practices (e.g., a Christian not attending church) than one emphasizing practices. Attitudes based on beliefs and practices also influence what struggles religious group members may face when they realize they no longer share certain beliefs or practices with the rest of the group. Although, some religious groups may accept a “doubting Thomas,” belief-oriented religions may have less tolerance for such dissent than religions where practice defines religiosity.

### Conflict of interest statement

The authors declare that the research was conducted in the absence of any commercial or financial relationships that could be construed as a potential conflict of interest.

## Footnotes

^1^Religiosity and spirituality may be conceptualized as two separate constructs. However, it was not our particular research interest to study the differences between self-reported religiosity and self-reported spirituality. To the extent that either of these offer indications of participants' adherence to particular sets of beliefs and/or practices, these constructs should be positively related to each other. Thus, this item may be seen as a rough composite of these two overlapping constructs.
