# Supplementary material for: Tolerating the “doubting Thomas”: how centrality of religious beliefs vs. practices influences prejudice against atheists
Source: Front Psychol. 2015 Sep 8;6:1352. doi: 10.3389/fpsyg.2015.01352 (PMC4561750; doi:10.3389/fpsyg.2015.01352)
Supplement: Supplementary file 1 [file DataSheet1.PDF]

Supplementary Material

Tolerating the "doubting Thomas": How centrality of religious beliefs versus practices influences anti-atheist prejudice

Jeffrey Hughes[1]*, Igor Grossmann[1], Adam B. Cohen[2]

[1]Department of Psychology, University of Waterloo, Waterloo, Ontario, Canada
[2]Department of Psychology, Arizona State University, Tempe, Arizona, United States

*** Correspondence:** Jeffrey Hughes, Department of Psychology, University of Waterloo, 200 University Ave. W, Waterloo, Ontario, N2L 3G1, Canada.
j4hughes@uwaterloo.ca

1. Additional Analyses – Study 1

1.1. Moderation of religiosity

Religiosity significantly moderated the effect of religious group, $F(2,286) = 6.04$, $p = .003$, $\eta^2 = .035$. More religious Protestants showed more prejudice toward atheists than did less religious Protestants, $\beta = .34$, $t(286) = 3.68$, $p < .001$. We also found this to be the case for Jews, $\beta = .26$, $t(286) = 2.16$, $p = .03$. However, Hindus showed no effect of religiosity on attitudes, $\beta = -.08$, $t(286) = -.95$, $p = .34$. At low levels of religiosity (-1 SD), no differences in attitudes between religions emerged other than a marginal difference between Jews and Hindus, $\beta = .19$, $t(286) = 1.89$, $p = .06$, such that Hindus showed marginally more negative attitudes. However, at high levels of religiosity (+1 SD), Protestants displayed significantly more prejudice than Hindus toward atheists, $\beta = -.34$, $t(286) = -4.03$, $p < .001$; while Jews fell in between both Protestants and Hindus, not significantly different from either (*p*s > .13).

These results provide evidence that religiosity moderates these results, although only Protestants and Hindus showed results in line with our expectations. Although here we find an effect of religiosity on attitudes for Jews, it should be noted that Jews in Study 2 do not show the same pattern. There, religiosity does not predict attitudes for Jews. This may be due to differences in the characteristics of Jews in these two samples. Study 1 gathered U.S. Jews from Mechanical Turk, whereas Study 2 randomly sampled Jews from a variety of countries, thus potentially being more representative of the population as a whole.

2. Attitudes Toward Atheists among Catholics – Study 2

Although our main focus was on religious groups with a strong emphasis on either beliefs or practices, here we extend our analyses to another dominant religious group, Catholics. Previous research has found Catholics fall between Protestants and Jews in their emphases on beliefs and

practices (Cohen and Hill 2007); however, whether this would similarly extend to attitudes toward atheists is an open question.

2.1. Method

All data were obtained from the 2008 ISSP: Religion III (ISSP Research Group 2012). We screened the ISSP sample for participants who identified as being Protestant (n = 12,188), Muslim (n = 2,167), Catholic (n = 22,982), Jewish (n = 1,104), or Hindu (n = 203).

As a measure of attitudes toward atheists, we used one item, "What is your personal attitude towards members of the following religious groups? Atheists or non-believers" (1="very positive" to 5="very negative").

We used a multilevel random intercepts model with participants (Level 1) nested in countries (Level 2). Religious group was dummy-coded, with Catholics as the comparison group. Because of differing sample sizes between groups leading to different within-group variances, we used a diagonal covariance matrix to model heterogeneous variance (Pinheiro and Bates 2000).

2.2. Results

We found a significant overall effect of religious group on attitudes toward atheists, $F(4,18506)$ = 13.36, $p < .001$, marginal $R^2 = .008$, conditional $R^2 = .194$. As Table S3 shows, Catholics did not differ in their attitudes from Protestants, $B = .03$, $t(18506) = 1.25$, $p = .21$, 95% CI [-.02, .08]; but had marginally less negative attitudes toward atheists compared to Muslims, $B = .15$, $t(18506) = 1.92$, p = .054, 95% CI [-.003, .30]; and had more negative attitudes compared to Jews, $B = -.47$, $t(18506)$ = -3.39, $p = .001$, 95% CI [-.74, -.20]; and Hindus, $B = -.65$, $t(18506) = -5.74$, $p < .001$, 95% CI [-.88, -.43]. These patterns held when simultaneously controlling for age, gender, and education, $F(4,18380) = 13.05$, $p < .001$.

Within this large, international dataset, we found that Catholics showed similarity with Protestants in their attitudes toward atheists, as one might expect given their considerable shared beliefs. They did, however, have slightly less negative attitudes compared to Muslims, suggesting tentative evidence that Catholics may be intermediate between highly belief-oriented religions (Islam) and practice-oriented religions (Judaism and Hinduism) in their attitudes toward atheists.

3. Additional Analyses – Study 3

Due to low reliability with the other four attitude items, we examined the disgust item separately. We found no significant participant religion × target religion × target beliefs interaction, $F(1,572) = 1.14$, $p = .29$, $\eta^2 = .002$, or participant religion × target religion × target practices interaction, $F(1,572)$ = .30, $p = .59$, $\eta^2 < .001$; nor were there any main effects.

References

Cohen, A. B., and Hill, P. C. (2007). Religion as culture: Religious individualism and collectivism among American Catholics, Jews, and Protestants. *Journal of Personality* **75**:4, 709–742. doi:10.1111/j.1467-6494.2007.00454.x

ISSP Research Group. (2012). International Social Survey Programme: Religion III - ISSP 2008. ZA4950 Data file version 2.2.0. Cologne: GESIS Data Archive. doi:10.4232/1.11334

Pinheiro, J. C., and Bates, D. M. (2000). *Mixed-effects models in S and S-PLUS*. New York: Springer.

Table S-1

Demographics for studies 1–3

	N	% Women	Age	% Completed college	Religiosity
Study 1					
Protestants	100	50.0%	38.2 (14.8)	58.0%	4.84 (1.71)
Jews	56	41.1%	31.8 (12.2)	63.6%	4.37 (1.79)
Hindus	150	37.3%	30.7 (9.6)	89.3%	5.23 (1.46)
Study 2					
Protestants	12,188	57.7%	48.7 (17.4)	34.8%	4.43 (1.35)
Muslims	2,167	50.6%	39.7 (14.7)	16.4%	5.37 (1.22)
Jews	1,104	56.5%	45.2 (17.9)	40.0%	4.02 (1.61)
Hindus	203	51.7%	43.0 (16.5)	26.6%	5.26 (1.11)
Study 3					
Protestants	311	42.8%	–	–	–
Jews	271	45.8%	–	–	–

Note. Due to a technical error, age and education were not collected for Study 3. Means and standard deviations (in parentheses) are displayed for age and religiosity. Religiosity was assessed on a 1–7 scale.

Table S-2

Religious composition of each ISSP country's sample

	N	Protestant	Muslim	Jewish	Hindu
Australia	1718	29.5%	.6%	.2%	.3%
Austria	1020	4.4%	2.4%	–	–
Belgium	1263	.7%	1.0%	.1%	–
Chile	1505	16.3%	.1%	.9%	–
Croatia	1201	.2%	1.0%	.1%	.1%
Cyprus	1000	.1%	–	–	–
Czech Republic	1512	4.0%	–	–	–
Denmark	2004	80.7%	.2%	–	–
Dominican Republic	2086	17.1%	.05%	.1%	–
Finland	1136	77.2%	–	–	–
France	2454	1.2%	.9%	.7%	–
Germany	1706	30.7%	2.8%	.1%	.3%
Hungary	1010	21.2%	.1%	.3%	–
Ireland	2049	2.5%	.5%	.1%	.05%
Israel	1193	.3%	9.5%	83.1%	–
Italy	1078	.4%	–	–	–
Japan	1200	–	–	–	–
Latvia	1069	21.2%	–	–	–
Mexico	1471	7.3%	.1%	.1%	–
Netherlands	1951	21.5%	1.4%	.2%	.4%
New Zealand	1027	33.2%	.4%	.4%	1.0%
Norway	1072	77.9%	.9%	–	–
Philippines	1200	1.4%	5.4%	–	–
Poland	1263	1.0%	–	–	–
Portugal	1000	2.6%	.2%	.1%	–
Russia	1015	.3%	4.3%	–	–
Slovak Republic	1138	11.8%	–	–	–
Slovenia	1065	1.4%	2.0%	–	–
South Africa	3292	49.6%	4.8%	.1%	4.3%
South Korea	1508	25.9%	–	–	–
Spain	2373	.9%	1.3%	–	–
Sweden	1235	65.6%	1.1%	.1%	.1%
Switzerland	1229	32.2%	3.0%	.6%	.2%
Taiwan	1927	4.0%	–	–	–
Turkey	1453	–	98.6%	–	–
Ukraine	2036	1.2%	.5%	.1%	–
United Kingdom	3075	38.7%	1.3%	.6%	.7%
United States	1365	51.0%	.7%	1.8%	.4%
Uruguay	1010	13.7%	–	.2%	–
Venezuela	1077	11.7%	–	–	–

Table S-3

Estimated means of negative atheist attitudes

	Protestants	Muslims	Jews	Hindus	Catholics
Mean	3.01	3.13	2.51	2.33	2.98
n	12,188	2,167	1,104	203	22,982
